# Differences in mental health symptoms and treatment by sexual orientation and migration background in a population-based sample

**DOI:** 10.1007/s00127-025-02848-w

**Published:** 2025-02-17

**Authors:** Andreas Malm, Petter Tinghög, Richard Bränström

**Affiliations:** 1 Department of Clinical Neuroscience, Karolinska Institutet, Nobels Väg 9, 171 77 Stockholm, Sweden; 2 Department of Health Sciences, Swedish Red Cross University, Hälsovägen 11, 141 57 Huddinge, Sweden

**Keywords:** Migrant, Sexual minority, Lesbian, Gay, Bisexual, Mental health, Minority stress, Intersectionality

## Abstract

**Purpose:**

Although sexual minorities are consistently found to be at excess risk of poor mental health, less is known about the mental health of individuals with dual minority statuses based on sexual orientation and migration background. This study aimed to examine prevalence of and disparities in mental health symptoms and treatment for common mental disorders (CMD) among sexual minority migrants; and to explore the potential mediating role of interpersonal and social stress.

**Methods:**

Participants were drawn from the Swedish Public Health Survey, 2018. The analytic sample included 104,652 individuals with complete records on all study variables (37.1%). The survey assessed mental health symptoms and interpersonal and social stress and was complemented with information on psychiatric treatment from comprehensive nationwide registries. Using logistic regression and mediation analyses, six groups were compared based on sexual orientation and migration background.

**Results:**

Greater risk of mental health symptoms was found among Swedish-born and non-European sexual minorities (adjusted odds ratios (OR) = 2.20, 95% confidence intervals (CI): 1.89–2.57, and OR = 2.10, 95% CI 1.34–3.29, respectively) compared to Swedish-born heterosexuals. Swedish-born sexual minorities were at greater risk of receiving treatment for CMD (OR = 2.58, 95% CI 2.20–3.01), while non-European heterosexuals showed lower risk (OR = 0.61, 95% CI 0.52–0.72). Perceived discrimination was less common among non-European sexual minorities compared to their Swedish-born counterparts and partially mediated the association between migration background and mental health symptoms.

**Conclusions:**

Sexual minority migrants are at greater risk of mental health symptoms compared to Swedish-born heterosexuals but not compared to Swedish-born sexual minorities. Providing mental health care for sexual minorities, including sexual minority migrants, and targeting sexual orientation discrimination, should be a priority.

**Supplementary Information:**

The online version contains supplementary material available at 10.1007/s00127-025-02848-w.

## Background

Global migration has continuously increased during the past five decades [1]. To ensure equal health for all, there is a need to monitor and understand the mental health situation among migrant populations [2]. Although some studies have reported higher rates of common mental health problems, e.g., depression and anxiety, among migrants compared to the general population [3, 4], there is also evidence suggesting that migrants in high-income countries have better health compared to the majority population of the host country [5]. A reason for these conflicting results is that migrants are a heterogenous group with large differences in health risk between different subgroups of migrants.

One subgroup of migrants that warrants particular interest is sexual minority individuals (i.e., non-heterosexuals) with a history of migration, given the increased risk of poor mental health and well-being consistently found among sexual minority individuals compared to heterosexuals [6–8]. According to minority stress theory, sexual minority individuals’ increased risk of poor mental health can be explained by this groups’ higher likelihood of being exposed to different types of stigma-based social stress, including discrimination, threats, and violence [9]. As an extension of the minority stress model, sexual minority individuals’ lower degree of social safety and social integration in terms of social connectedness, belonging, and inclusion, has been suggested as important factors linking stigma and poor mental health [10, 11]. In line with this, social support has been suggested to function as a buffer against the negative impact from minority stress factors [9, 12]. Furthermore, low social support is associated with increased risk of poor mental health in certain migrant populations [13, 14], including sexual minority migrants [15].

Intersectionality theory posits that individuals with dual marginalized minority statuses are exposed to unique challenges due to their intersecting social identities [16], which in turn requires these interdependent statues to be taken into consideration jointly rather than separately when studying groups with several intersecting minority statuses [17]. Minority status groups may include, but are not limited to, sexual and gender minorities, migrants, and racialized group, with racialization referring to the social process of categorizing and marginalizing individuals based on characteristics such as color of skin or hair, language, or religion [18, 19]. Indeed, experiences of both racism and perceived heterosexism have been linked to symptoms of depression and anxiety among racialized sexual minorities in the US [20–22]. However, several population-based studies have found no excess risk of poor mental health among sexual minority individuals with migration background compared to their native counterparts [23–25].

Although symptoms of poor mental health are more prevalent in certain migrant populations [26, 27], migrants typically display lower rates of treatment for common mental disorders (CMD), including mood and anxiety disorders, compared to population groups without a history of migration [28–30], at least during the first decade after immigration [31]. However, there are differences in mental health care utilization among migrant subgroups with regard to length of stay in the host country, region of origin, reason for immigration, and type of care [31, 32]. Several reasons for the lower rate of treatments for CMD among migrants have been suggested, including: language difficulties, lack of awareness about the health care system, differences in help-seeking behavior, and greater stigma relating to mental health in some migrant groups [4, 33, 34]. Among sexual minority individuals, however, there is ample evidence for higher rates of mental health care utilization compared to heterosexuals [8, 35, 36].

The current study aims to investigate the prevalence of mental health symptoms and treatment for CMD among sexual minority migrants of European and non-European origin in Sweden, and potential differences in these outcomes compared to heterosexuals without a history of migration. It further aims to investigate prevalence of and disparities in interpersonal and social risk factors, including perceived discrimination, victimization, and lack of social support and social trust. Its final aim is to investigate the potential mediating role of these risk factors on the associations between minority status and mental health symptoms and treatment for CMD. Based on the theoretical framework of minority stress, social safety, and intersectionality, we expect to see the greatest risk of mental health symptoms among the dual minority status holders, i.e., sexual minority migrants, in particular those born outside of Europe, compared to Swedish-born heterosexuals. We further hypothesize that the greater risk of mental health symptoms will correspond to greater risk of risk of having received treatment for CMD in all sexual minority groups. Furthermore, we expect to find higher levels of interpersonal and social stress in all minority groups, in particular among the non-European sexual minorities. Finally, we hypothesize the associations between minority status and mental health symptoms and treatment for CMD to be mediated by interpersonal and social stress.

## Methods

### Participants

This study used participants from the Swedish Public Health Survey in 2018, a nationwide survey distributed by the Public Health Agency of Sweden in February 2018 [37]. An invitation to participate in the study was sent to a random sample of 282,086 individuals. Participants were offered the option to respond via either paper-and-pencil mailed questionnaires or self-administered web surveys. A total of 117,178 individuals returned the survey (41.5% of those invited). Our analytic sample included 104,652 individuals with complete records on all study variables (37.1%). The survey assessed, among other things, sociodemographic background, sexual orientation, mental health symptoms, and social determinants of mental health. The survey was supplemented with data from nationwide registries regarding age, legal gender, level of education, income, urbanicity, and country of birth. Using the personal identification numbers available in Sweden, data on physician-assessed psychiatric diagnoses and the use of prescribed psychiatric medication were linked to survey responses using two different comprehensive nationwide registries: the National Patient Register, and the National Prescribed Drug Register. From the National Patient Register, which contains information on all specialized outpatient and inpatient health care visits, data on all psychiatric diagnoses given between February 1, 2018, and December 31, 2018, was retrieved. Data on the use of prescribed psychiatric medication between February 1, 2018, and December 31, 2018, was collected from the Swedish Prescribed Drug Register, which contains information on all prescribed and purchased medication. The study was approved by the Regional Ethics Committee in Stockholm (No. 2013/2200–31/2; No. 2019–06335).

### Measures

#### Sociodemographic covariates

Sociodemographic factors included nationally registered gender (only two genders are currently registered in Sweden: male and female), age (16–25, 26–35, 36–45, 46–55, 56–65, 66–84 years), level of education (≤ 9 years, ≥ 10 years no university degree, university degree), income (below the risk of poverty threshold, which is 60% of the national median equivalized disposable income, above the risk of poverty threshold), relationship status (living with partner, not living with partner), and urbanicity (larger city, smaller city, rural community). To facilitate the pairwise comparison of all included exposure groups, level of education was dichotomized as either having a university degree or not, and urbanicity was dichotomized as either living in a larger city or not (i.e., living in a smaller city or in a rural community).

#### Sexual orientation and migration background

*Sexual orientation.* All individuals were categorized as either heterosexual or sexual minority, based on self-identification using the following question “What is your sexual orientation?”, to which five response options were given: “heterosexual”, “homosexual”, “bisexual”, “other”, or “I don’t know”. For respondents who reported “other” sexual orientation, there was an open-ended option with the instruction “*please specify*”, which was in fact used by respondents of all categories. In addition to identification as heterosexual, bisexual, or homosexual, the most common “other” sexual orientation reported was: pansexual (*n* = 113), asexual (*n* = 61), and queer (*n* = 23). Those who responded “I don’t know” (*n* = 256) were categorized as sexual minority if they indicated sexual minority status to the open-ended response option, otherwise they were categorized as heterosexual.

*Migration background.* Individuals’ migration background was categorized based on registry data on country of birth, grouped into larger geographic regions: born in Sweden, born in a European country other than Sweden, and born outside of Europe. Based on the concept of racialization, the group born outside of Europe was assumed to consist of individuals who were more likely to have been exposed to racialization in a Swedish context compared to those born in another European country, based on their appearance (including color of skin and hair, but also clothing and religious markers). The five most common non-European countries of birth for the Swedish population are Syria, Iraq, Iran, Somalia, and Afghanistan [38]. However, given that the countries included in the non-European category are very diverse, the sample was assumed to be heterogenous with regard to negative experiences of racialization.

*Intersectional minority status*. Based on sexual orientation and migration background, six groups with combinations of these two statuses were created: heterosexual individuals born in Sweden, heterosexual individuals born in Europe, heterosexual individuals born outside of Europe, sexual minority individuals born in Sweden, sexual minority individuals born in Europe, and sexual minority individuals born outside of Europe.

#### Risk and protective factors

*Interpersonal and social stress.* Interpersonal and social stress exposure was conceptualized based on the minority stress and social safety models [9, 11], including experiences of perceived discrimination, victimization/threats of victimization, and lack of social support and social trust. Some of the variables described below have been used in previous studies among sexual minorities [8].

*Perceived discrimination* was assessed using the item: “During the past three months, have you been treated in a way that made you feel discriminated against?”, with the response options “No”, “Yes, sometimes”, and “Yes, several times”. Responses were dichotomized as “No” and “Yes”. *Victimization* was assessed using two items, one concerning physical violence (“During the past 12 months, have you been subjected to physical violence?”, with response options “Yes”/”No”), and one concerning threat or threats of violence (“During the past 12 months, have you been subjected to threat or threats of violence, so that you were scared?”, with response options “Yes”/”No”). A positive response to any or both items was coded as a positive response for victimization.

Social support was assessed using two items, covering emotional and instrumental social support. For emotional social support, the following item was used: “Do you have anyone you can share your innermost feelings with and confide in?”, with the response options “Yes”/”No”. For instrumental social support, the following item was used: “Can you get help from any person if you have practical problems *or* are ill?”, with the response options “Yes, always”, Yes, most of the time”, “No, mostly not”, and “No, never”. Responses were dichotomized as “Yes” (always/most of the time) and “No” (mostly not/never). *Low social support* was operationalized as lacking either emotional or instrumental social support, or both. The item “Do you think that, in general, people can be trusted?” was used to assess social trust, with response options “Yes”/”No”. Participants were classified as having *low social trust* if they gave a negative response.

#### Outcomes

*Mental health symptoms.* Self-rated symptoms of poor mental health were assessed using a five-item version of the General Health Questionnaire [39], the GHQ-5. The 12-item version of the GHQ has been widely used in epidemiological research and has been validated in the general Swedish population [40]. Furthermore, it has shown satisfactory sensitivity and specificity for predicting current diagnosis of depression [41, 42]. The GHQ-5 assesses mental health symptoms in the past few weeks (unhappy or depressed mood, loss of confidence in self, constantly feeling tense, feelings of worthlessness, and feeling of not being able to overcome difficulties). Consistent with prior literature [42], a dichotomous variable was created, with a total score of ≥ 2 to denote current mental health symptoms and a score of ≤ 1 to denote no current mental health symptoms.

*Treatment for common mental disorders*. Information on psychiatric diagnoses from specialized outpatient and inpatient health care visits and the use of prescribed psychiatric medication was retrieved from comprehensive nationwide registries. Each health care visit had been coded with a primary diagnosis from the International Statistical Classification of Diseases and Related Health Problems, Version 10 (ICD-10) [43], by the treating physician. In addition to the primary diagnosis, up to 20 supplementary ICD-10 diagnostic codes could be set for each visit. Respondents were coded as having received treatment for CMD if they had received a diagnosis for at least one of the included mood or any anxiety and stress-related disorders (ICD-10: F32-F34, F40-F43). Respondents were also classified as having received treatment for CMD if they had received prescribed psychiatric medication corresponding to these diagnoses, including antidepressants, anxiolytic, and hypnotics and sedatives (N06A, N05B, and N05C according to Anatomical Therapeutic Chemical Classification System). Participants who had neither received a CMD diagnosis nor psychiatric medication were categorized as having received no treatment for CMD.

### Statistical analyses

In all analyses described below, weights were used to adjust for selection probabilities and non-response to generate nationally representative estimates of prevalence and associations. The sociodemographic distribution of the sample and differences in sociodemographic characteristics based on sexual orientation and migration background were examined using descriptive statistics. Differences were assessed using chi-square and t-tests. For all groups based on sexual orientation and migration background, prevalence estimates for outcomes and risk factors were calculated. In a series of logistic regression analyses, sexual orientation and migration background-based differences in mental health symptoms and treatment for CMD, and in interpersonal and social risk factors, were calculated.

We then examined effect modification by sexual orientation and migration background, i.e., whether the associations between interpersonal and social risk factors and the outcomes differed by sexual orientation and/or migration background. A significant interaction for sexual orientation or migration background and the interpersonal and social risk factors was interpreted as evidence of differences based on sexual orientation and migration background, respectively, in the association between these risk factors and mental health symptoms and treatment for CMD. A significant interaction for sexual orientation and the interpersonal and social risk factors would motivate further mediation analyses stratified by sexual orientation, while a significant interaction for migration background and the proposed risk factors would motivate a separation of the migrant groups in the further analyses.

In analyses stratified by sexual orientation, we then examined whether the proposed interpersonal and social risk factors mediated the association between migration background and mental health symptoms and treatment for CMD using multiple mediation analyses. All four proposed mediating variables were included in the multiple mediation analyses. To statistically test mediation, we calculated the indirect effect of each variable as a mediator between migration background and mental health symptoms and treatment for CMD.

In addition to crude analyses, analyses were also adjusted for gender, age, education, income, relationship status, and urbanicity. Analyses were supplemented by robust 95% confidence intervals (CIs) for prevalence rates and odds ratios (ORs), respectively. Data analyses were conducted using SPSS (Version 28) and Mplus (Version 8). Mediation analyses were performed in Mplus, using maximum likelihood parameter estimates with robust standard errors (MLR) to calculate direct and indirect effects with 95% CIs. A significant indirect effect (*p* < 0.05) was interpreted as evidence of mediation.

## Results

### Descriptive statistics

Table 1 presents sociodemographic characteristics of the sample stratified by sexual orientation and migration background. Groups were compared to the reference group of heterosexual individuals born in Sweden. Although similarities in sociodemographic characteristics were found among all minority groups, there were also several notable differences between the groups. For instance, sexual minority individuals born in Sweden, Europe and outside of Europe were more likely to be younger, having a lower income, living in a larger city, and living without a partner compared to heterosexual individuals born in Sweden. Furthermore, Swedish- and European-born sexual minority individuals were more likely to be having a university degree, while sexual minority individuals born in Sweden were more likely to be female. Additional information and pairwise comparisons between all groups are presented in Online Resource 1.

**Table 1:** Sociodemographic characteristics of the participants in the National Health Survey, 2018, by sexual orientation and migration background

	Heterosexual			Sexual minority		
	Swedish	European	Non-European	Swedish	European	Non-European
Total* n (%),* unweighted	90 428 (86.4)	6802 (6.5)	4188 (4.0)	2766 (2.6)	235 (0.2)	233 (0.2)
	*%*^*a*^	*%*^*a*^	*%*^*a*^	*%*^*a*^	*%*^*a*^	*%*^*a*^
Gender						
Male	51.2	46.3	48.9	39.2	54.7	50.0
Female	48.8	53.7	51.1	60.8	45.3	50.0
Age, years						
Mean	48.8	53.2	42.3	32.3	42.3	34.4
16–25	14.7	6.5	16.9	43.9	15.0	33.2
26–35	14.5	12.9	20.7	24.9	32.7	22.6
36–45	15.2	15.7	23.2	13.8	15.5	25.9
46–55	15.2	16.3	17.4	7.8	11.5	9.5
56–65	16.9	18.4	12.4	5.4	10.6	7.1
66–84	23.5	30.2	9.3	4.3	14.7	1.8
Level of education						
≤ 9 years	18.8	17.5	24.4	26.4	8.4	22.9
≥ 10 years, no university degree	44.3	37.2	27.1	33.2	39.2	34.6
University degree	36.0	40.8	39.9	38.9	49.5	37.0
Missing	0.9	4.6	8.6	1.4	3.0	5.5
Income						
Below poverty line^b^	20.7	25.9	44.3	48.9	34.2	55.6
Relationship status						
Living with partner	63.5	65.7	55.4	35.7	53.3	45.0
Not living with partner	36.5	34.3	44.6	64.3	46.7	55.0
Urbanicity						
Larger city	30.8	43.5	49.3	39.0	46.2	58.5
Smaller city	34.7	29.8	29.7	35.1	29.4	25.4
Rural community	34.5	26.7	21.0	26.0	24.4	16.1

^a^Weighted percentages

^b^Below 60% of median income

### Prevalence of mental health symptoms and treatment for common mental disorders

**Fig. 1:**
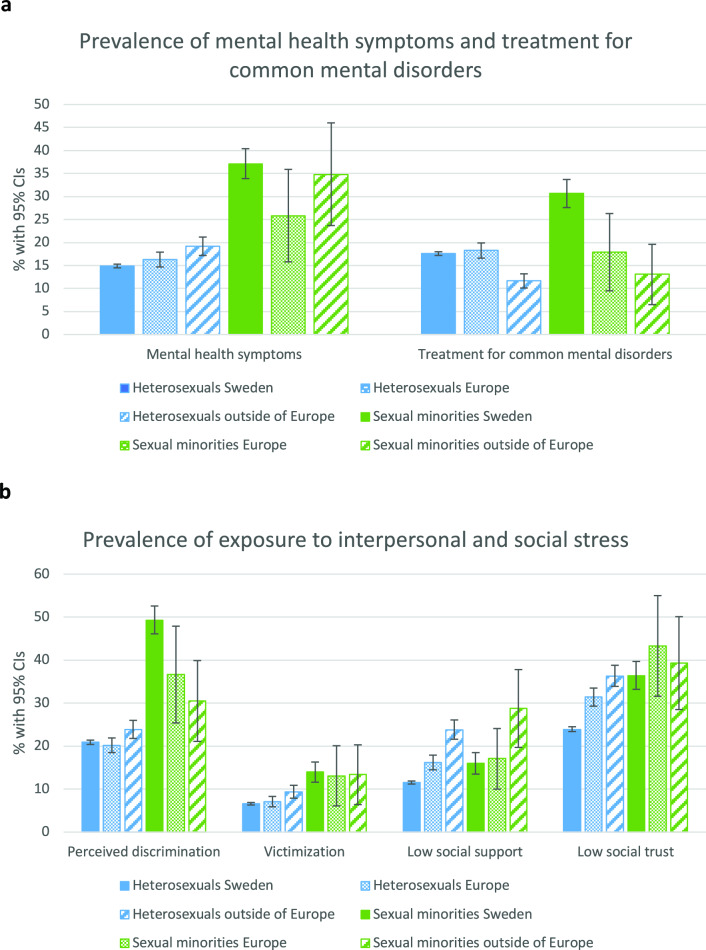
**a** Prevalence of mental health symptoms and treatment for common mental disorder by sexual orientation and migration background. Prevalence is shown in percent with 95% confidence intervals (CIs), **b** Prevalence of exposure to interpersonal and social stress by sexual orientation and migration background. Prevalence is shown in percent with 95% confidence intervals (CIs)

Figure 1a presents prevalence estimates of mental health symptoms and treatment for CMD stratified by sexual orientation and migration status. In general, sexual minority individuals reported higher levels of mental health symptoms than heterosexual individuals, with the highest prevalence found among the Swedish born sexual minority individuals. When viewing the prevalence of treatment for CMD, a somewhat different pattern emerged, with lower rates of treatment found among heterosexual individuals of non-European origin. Again, the highest rates were found among sexual minority individuals born in Sweden.

#### Prevalence of interpersonal and social stress exposure

Figure 1b shows the prevalence of interpersonal and social stress exposure stratified by sexual orientation and migration background. Interpersonal stress exposure in the form of perceived discrimination and victimization followed the same pattern, with heightened prevalence found in particular among sexual minority individuals born in Sweden. Exposure to social stress appears to follow a somewhat different pattern, with low social support found in all minority groups, although in particular among the non-European migrants. Prevalence of low social trust was heightened in all minority groups.

#### Sexual orientation and migration background-based disparities in mental health symptoms and treatment for common mental disorders

Table 2 presents adjusted odds ratios (ORs) for sexual orientation and migration background-based disparities in mental health symptoms and treatment for CMD. Greater risk of mental health symptoms was found in all sexual minority groups (born in Sweden: OR = 2.20, 95% CI 1.89–2.57; born in Europe: OR = 1.67, 95% CI 1.01–2.79; born outside of Europe: OR = 2.10, 95% CI 1.34–3.29), as well as among the European-born heterosexual individuals (OR = 1.18, 95% CI 1.03–1.34).

**Table 2:** Adjusted odds ratios for sexual orientation and migration background-based disparities in mental health symptoms and treatment for common mental disorders

	Mental health symptoms OR (95% CI)	Treatment for common mental disorders OR (95% CI)
Heterosexuals born in Sweden (reference)	1	1
Heterosexuals born in Europe	1.18 (1.03–1.34)*	0.90 (0.80–1.01)
Heterosexuals born outside of Europe	1.09 (0.95–1.25)	0.61 (0.52–0.72)*
Sexual minorities born in Sweden	2.20 (1.89–2.57)*	2.58 (2.20–3.01)*
Sexual minorities born in Europe	1.67 (1.01–2.79)*	1.09 (0.63–1.91)
Sexual minorities born outside of Europe	2.10 (1.34–3.29)*	0.82 (0.46–1.48)

OR, Odds ratios; CI, confidence intervals

All analyses are adjusted for gender, age, educational level, income (below poverty line), relationship status and urbanicity, with weights applied

*Significant at *p* < 0.05

Greater risk of having received treatment for CMD was found among the Swedish-born sexual minority individuals (OR = 2.58, 95% CI 2.20–3.01). Interestingly, lower risk was found in the non-European heterosexual group (OR = 0.61, 95% CI 0.52–0.72), thus pointing in the opposite direction compared to ORs for mental health symptoms in this group. All other results were non-significant.

#### Effect modification by sexual orientation and migration background

Interaction effects are shown in Online Resource 2. Sexual orientation was found to be a significant effect modifier of social support in the association with mental health symptoms (ß = −0.403, *p* < 0.05), and of social trust in the association with treatment for CMD (ß = 0.333, *p* < 0.05), indicating that the association between interpersonal and social stress exposure and mental health symptoms and treatment for CMD differed by sexual orientation. Migration background was found to be a significant effect modifier of social trust in the association with treatment for CMD (ß = −0.383, *p* < 0.05), but only for migrants born outside of Europe, indicating that there are different experiences for migrants of European and non-European origin that motivate the separation of these groups in the analyses.

#### Mediators of migration background-based disparities in mental health symptoms and treatment for common mental disorders stratified by sexual orientation

**Fig. 2:**
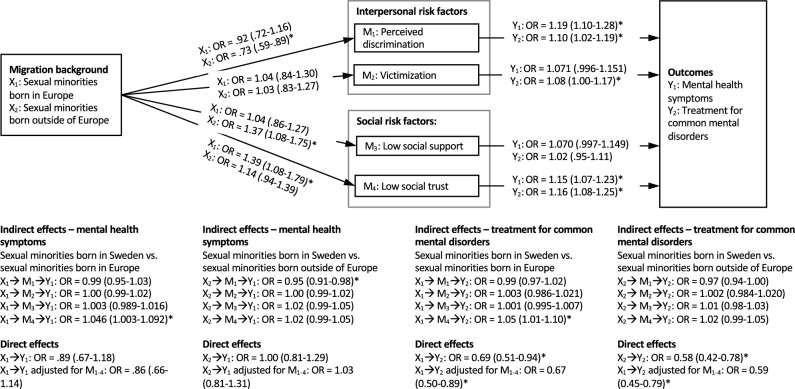
Indirect effect of migration background on mental health symptoms and treatment for common mental disorders among sexual minorities, through interpersonal and social mediators. *OR* odds ratios with 95% confidence intervals. *Significant at *p* < 0.05

**Fig. 3:**
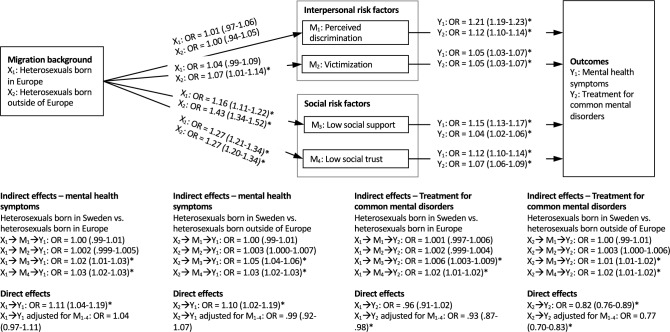
Indirect effect of migration background on mental health symptoms and treatment for common mental disorders among heterosexuals, through interpersonal and social mediators. *OR* odds ratios with 95% confidence intervals. *Significant at *p* < 0.05

Based on the findings that the association between interpersonal and social stress exposure and mental health symptoms and treatment for CMD differed by sexual orientation, analyses of the potential mediating role of interpersonal and social stress on these associations stratified by sexual orientation were performed. Direct and indirect effects for sexual minority individuals are shown in Fig. 2, and for heterosexual individuals in Fig. 3.

For sexual minority individuals, no significant direct effects of migration status on mental health symptoms were found for either migrant group. Among those born in Europe, a significant indirect mediating effect for low social trust in the association between migration status and mental health symptoms (OR = 1.046, 95% CI 1.003–1.092) and treatment for CMD (OR = 1.05, 95% CI 1.01–1.10) was found. This indicates that the association between migration status and treatment for CMD was partially mediated by low social trust among migrants of European origin. Among sexual minority individuals born outside of Europe, however, the only significant indirect mediating effect found was for perceived discrimination in the association between migration status and mental health symptoms (OR = 0.95, 95% CI 0.91–0.98).

Among heterosexual orientation individuals, however, a different pattern emerged. For those of European origin, a significant indirect mediating effect for low social support and low social trust in the association between migration status and both mental health symptoms (OR = 1.02, 95% CI 1.01–1.03 and OR = 1.03, 95% CI 1.02–1.03, respectively) and treatment for CMD (OR = 1.006, 95% CI 1.003–1.009 and OR = 1.02, 95% CI 1.01–1.02, respectively) was found. The same pattern was seen for heterosexual individuals of non-European origin, with a significant indirect mediating effect for low social support and low social trust in the association between migration status and both mental health symptoms (OR = 1.05, 95% CI 1.04–1.06 and OR = 1.03, 95% CI 1.02–1.03, respectively) and treatment for CMD (OR = 1.01, 95% CI 1.01–1.02 and OR = 1.02, 95% CI 1.01–1.02, respectively). Furthermore, the association between migration status and mental health symptoms was no longer significant with the mediating variables included in the model. This finding suggests that the elevated levels of mental health symptoms reported by heterosexual migrants are explained by lower levels of social support and social trust.

## Discussion

In this population-based study we found elevated prevalence of mental health symptoms among both Swedish-born sexual minorities and sexual minority individuals with a history of migration. While the higher prevalence of mental health symptoms among sexual minority individuals is in line with evidence from previous research [6–8], this study extends previous findings by showing that the increased risk of mental health symptoms among sexual minority individuals is similarity elevated among both sexual minorities with a history of migration and those without such experience. In contrast, the similarity in elevated mental health symptoms across groups was not reflected in a similarly elevated use of treatment for CMD. While sexual minority individuals with a history of migration were much more likely to report mental health symptoms compared to heterosexuals, they were no more likely than heterosexuals to have received treatment for CMD. Mediation analyses showed that different interpersonal and social risk factors mediated the association between migration background and mental health symptoms and treatment for CMD depending on sexual orientation.

This study does not suggest an excess risk of mental health symptoms among individuals with a dual minority status, i.e., both as an immigrant and as a sexual minority individual. This finding is contrary to our hypothesis that the dual minority status groups would display the greatest risk of mental health symptoms. This may seem counter-intuitive from an intersectionality perspective and contradicts some studies indicating an increased health risk among those with multiple stigmatized statuses [44]. Some studies have suggested that having multiple marginalized minority statuses impose a multiplied risk of being subjected to prejudice and other stigma-related adverse experiences [45], factors known to be associated with increased risk of poor mental health among minorities [9]. Our findings contradict such patterns, and are in line with findings from several recent population-based studies in the Swedish context [23–25], that, taken together, highlight the importance of not assuming that multiple stigmatized minority statuses automatically lead to additional and multiplied risk of poor mental health.

ln discordance with our hypothesis, the risk of treatment for CMD was not increased for the sexual minority migrant groups. While this risk was indeed increased among the Swedish-born sexual minorities, the absence of excess risk in the dual minority status groups could possibly be at least partially related to certain migrant subgroups’ lower health care utilization [31]. Whether this is the case in our study is however not possible to determine.

There are likely several factors that contribute to the finding that no excess risk of mental health symptoms was found among the sexual minority migrants compared to their Swedish-born counterparts. As the analyses stratified by sexual orientation show, the lower risk of mental health symptoms among the non-European sexual minority migrants is partly explained by lower levels of perceived discrimination in this group, compared to their Swedish-born counterparts. This finding is contrary to our hypothesis that the non-European sexual minority migrants would show the highest levels of perceived discrimination. This may indeed also seem counter-intuitive, as discrimination based on both sexual orientation and racialization has been reported in dual minority status groups [46]. However, as the majority of non-European migrants come from countries characterized by high stigma towards sexual minorities, they may have developed coping strategies adjusted to high-stigma environments, e.g., concealing their sexual orientation, a strategy that has been shown to be protective against experiences of discrimination and victimization in high-stigma settings [47]. The lower risk of discrimination reported by non-European migrants could possibly also reflect the heterogeneity of this group: although many non-European migrants in Sweden come from countries in the Middle East and Africa, respondents in this group also originate from other parts of Asia, North and South America, Oceania, and the former Soviet Union, and experiences of racialization-based discrimination may vary accordingly. Furthermore, according to the intersectional invisibility theory, certain advantages may come from being doubly disadvantaged; individuals with intersecting marginalized identities are seen as non-prototypical members of their respective subordinate groups and may thus face less direct oppression, including discrimination, compared to more prototypical group members [48].

While the understanding of the effects of migrating between different-level stigma locales is still limited, some studies suggest that country-of-origin structural stigma may continue to negatively affect the health of sexual minority migrants even after relocating to a low-structural stigma context like Sweden [49], at least among older individuals and in the first ten years after migration [50]. It is possible that sexual minority individuals who have moved from high-stigma countries perceive their new environment in Sweden as less stigmatizing and more accepting [51]. Whether this is true for the non-European sexual minority individuals in our study remains unknown, but this could contribute to explaining the lower levels of perceived discrimination among the non-European sexual minority migrants compared to their Swedish-born counterparts.

Results from the mediation analyses stratified by sexual orientation show a more complex pattern than what we had hypothesized. The analyses suggest that different factors relating to interpersonal and social stress appear to be linked to mental health for sexual minority and heterosexual individuals, respectively. Although no significant direct effect between migration background and mental health symptoms was found for sexual minority individuals, this association was partially mediated by low social trust among the European sexual minority migrants, while the non-European migrants’ lower risk of perceived discrimination partially mediated the association between migration status and mental health symptoms. For heterosexuals, however, the association between migration status and mental health symptoms was mediated by low social support and low social trust, which fully explained the greater risk of mental health symptoms among heterosexual migrants of European origin compared to heterosexual natives. In contrast, the inclusion of the potential mediating factors in the model only slightly affected the association between migration status and treatment for CMD, for heterosexual and sexual minority individuals alike. Thus, the lower likelihood of having received treatment for common mental disorders found among heterosexuals of non-European origin is likely due to factors that have not been considered in the current study. Whether these include potential barriers to mental health care utilization [33, 34] cannot be determined from our findings, but this should be further investigated in future studies.

### Strengths and limitations

Although the current study has severable notable strengths, including a large national population-based representative sample and the use of comprehensive measures of key components of minority stress, results should be considered in the light of several limitations. First, the cross-sectional design of the study with simultaneous assessment of interpersonal and social stress exposure and mental health symptoms prevents us from establishing the causal direction of those associations. However, the longitudinal follow-up of treatment for CMD after completion of the survey using registry data provided an opportunity to temporally test the link between reports of interpersonal and social stress on future mental health treatments. However, we do not have information on what type of treatment those with psychiatric diagnoses received, which is a limitation. Second, the assessment of mental health symptoms relied on self-report. While a diagnostic interview might have yielded other results, we tried to compensate for this by also including information on treatment and physician-assed psychiatric diagnoses. Third, some of the included variables were based on single item measures. While single item measures have been criticized for being less reliable and for not capturing nuances, the use of single item measures is not necessarily inferior to using multiple item measures. By using single item measure, it is often possible to investigate a broader range of factors, without overburdening the respondents [52]. Fourth, despite having an acceptable response rate, the current study also contained a substantial number of non-responders. The fact that the survey was available in Swedish and English only could have affected the response rate among migrants. While many non-European migrants in Sweden have refugee background, higher response rate among non-European migrants could have yielded higher prevalence of mental health symptoms in this group, as refugees typically show higher prevalence of mental health symptoms compared to natives and other immigrants [3, 53, 54]. However, it seems unlikely that sexual orientation-based disparities in mental health would be affected by potential language barriers, as these would have the same effect on response rate regardless of sexual orientation. Furthermore, calibration weights were used to make the population representative of the total population in Sweden. Fifth, it cannot be ruled out that some respondents may have chosen not to disclose their sexual minority status. Since sexual orientation concealment is more likely to occur in countries with discriminatory laws towards and low acceptance for sexual minorities [55], sexual minority migrants may be overrepresented among the non-disclosers. Although under-reporting of sexual minority status among sexual minority migrants could lead to distorted estimates of risk of mental health symptoms and treatment for CMD for these groups, it seems unlikely that potential under-reporting of sexual orientation would have affected estimates in any significant way. Finally, it is possible that excess risk of poor mental health would have been found for certain subgroups among migrants, for instance with regard to reason for migration (i.e., voluntary or forced) or type of residence permit (temporary or permanent), if we had been able to investigate this. However, as we did not have access to this information, we cannot draw any conclusions regarding subgroups among migrants, but the question should be addressed in future studies.

## Conclusions

While high prevalence of mental health symptoms was found among sexual minority individuals, our study found no excess risk of poor mental health among those with dual minority statuses based on sexual orientation and migration background compared to Swedish-born sexual minorities. However, with increased risk of mental health symptoms found among all sexual minority groups, providing mental health care for sexual minorities, including sexual minority migrants, should be a priority. The high levels of experiences relating to interpersonal stress found among sexual minority individuals point to the need for interventions on societal level to target sexual orientation-based discrimination and victimization, in order to improve mental health among sexual minority individuals.

## Supplementary Information

Below is the link to the electronic supplementary material.

Supplementary file1 (DOCX 89 kb)

Supplementary file2 (DOCX 24 kb)

## Data Availability

The statistical code is available from the corresponding author. Under Swedish law and ethical approval, individual level data of this kind cannot be publicly available. Individual level data can be made available on reasonable request as long as it is in line with Swedish law and ethical approvals.
