# Supplementary material for: Differences in mental health symptoms and treatment by sexual orientation and migration background in a population-based sample
Source: Soc Psychiatry Psychiatr Epidemiol. 2025 Feb 17;60(5):1197–209. doi: 10.1007/s00127-025-02848-w (PMC12119674; doi:10.1007/s00127-025-02848-w)
Supplement: Supplementary file 1 — Supplementary file1 (DOCX 89 kb) [file 127_2025_2848_MOESM1_ESM.docx]

**Online Resource 1**

**Title** Differences in mental health symptoms and treatment by sexual orientation and migration background in a population-based sample

**Journal** Social Psychiatry and Psychiatric Epidemiology

**Authors** Andreas Malm, Petter Tinghög, Richard Bränström

**Corresponding author** Andreas Malm ([andreas.malm@ki.se](mailto:andreas.malm@ki.se)), Department of Clinical Neuroscience, Karolinska Institutet, Nobels väg 9, 171 77 Stockholm, Sweden

**Online Resource 1** Sociodemographic characteristics of the participants in the National Health Survey, 2018, compared pairwise based on sexual orientation and migration background

|  | Heterosexual | Sexual minority | *X^2^* | F | Sig (p-value) |
| --- | --- | --- | --- | --- | --- |
| Total *n (%),* unweighted | 101 418 (96.9) | 3234 (3.1) |  |  |  |
|  | *%^a^ or M^b^* | *%^a^ or M^b^* |  |  |  |
| *Gender* |  |  | 120.1 |  | <0.001 |
| Male | 50.5 | 42.1 |  |  |  |
| Female | 49.5 | 57.9 |  |  |  |
| *Age, years* (mean) | 48.5 | 33.5 |  | 2788.95 | 0.000 |
| *Age groups, years* |  |  |  |  |  |
| 16-25 | 14.2 | 39.8 |  |  |  |
| 26-35 | 15.0 | 25.3 |  |  |  |
| 36-45 | 16.1 | 15.6 |  |  |  |
| 46-55 | 15.5 | 8.3 |  |  |  |
| 56-65 | 16.6 | 6.1 |  |  |  |
| 66-84 | 22.6 | 4.9 |  |  |  |
| *University degree* |  |  | 13.61 |  | <0.001 |
| Yes | 36.9 | 39.6 |  |  |  |
| *Income* |  |  | 1400.80 |  | <0.001 |
| Below poverty line^c^ | 23.8 | 48.5 |  |  |  |
| *Relationship status* |  |  | 1059.77 |  | <0.001 |
| Living with partner | 62.8 | 38.6 |  |  |  |
| *Urbanicity* |  |  | 127.35 |  | <0.001 |
| Larger city | 34.1 | 42.3 |  |  |  |
| Smaller city or rural community | 65.9 | 57.7 |  |  |  |

^a^ Weighted percentages

^b^ Mean value for age in years only

^c^ 60% of the national median equivalized disposable income

|  | Swedish-born | Migrant | *X^2^* | F | Sig (p-value) |
| --- | --- | --- | --- | --- | --- |
| Total *n (%),* unweighted | 93 194 (89.1) | 11 458 (10.9) |  |  |  |
|  | *%^a^ or M^b^* | *%^a^ or M^b^* |  |  |  |
| *Gender* |  |  | 53.95 |  | <0.001 |
| Male | 50.7 | 47.9 |  |  |  |
| Female | 49.3 | 52.1 |  |  |  |
| *Age, years (mean)* | 48.1 | 46.9 |  | 66.31 | <0.001 |
| *Age, years* |  |  |  |  |  |
| 16-25 | 15.9 | 12.7 |  |  |  |
| 26-35 | 14.9 | 17.5 |  |  |  |
| 36-45 | 15.1 | 19.8 |  |  |  |
| 46-55 | 14.9 | 16.6 |  |  |  |
| 56-65 | 16.4 | 14.9 |  |  |  |
| 66-84 | 22.7 | 18.5 |  |  |  |
| *University degree* |  |  | 132.86 |  | <0.001 |
| Yes | 36.1 | 40.4 |  |  |  |
| *Income* |  |  | 1901.90 |  | 0.000 |
| Below poverty line^c^ | 21.8 | 36.2 |  |  |  |
| *Relationship status* |  |  | 51.19 |  | <0.001 |
| Living with partner | 62.3 | 59.6 |  |  |  |
| *Urbanicity* |  |  | 1877.64 |  | 0.000 |
| Larger city | 31.2 | 46.9 |  |  |  |
| Smaller city or rural community | 68.8 | 53.1 |  |  |  |

^a^ Weighted percentages

^b^ Mean value for age in years only

^c^ Sixty percent of the national median equivalized disposable income

|  | Heterosexual born in Sweden | Heterosexual born in Europe | *X^2^* | F | Sig (p-value) |
| --- | --- | --- | --- | --- | --- |
| Total *n (%),* unweighted | 90 428 (86.4) | 6802 (6.5) |  |  |  |
|  | *%^a^ or M^b^* | *%^a^ or M^b^* |  |  |  |
| *Gender* |  |  | 81.28 |  | <0.001 |
| Male | 51.2 | 46.3 |  |  |  |
| Female | 48.8 | 53.7 |  |  |  |
| *Age*, years (mean) | 48.8 | 53.2 |  | 483.32 | <0.001 |
| Age*, years* |  |  |  |  |  |
| 16-25 | 14.7 | 6.5 |  |  |  |
| 26-35 | 14.5 | 12.9 |  |  |  |
| 36-45 | 15.2 | 15.7 |  |  |  |
| 46-55 | 15.2 | 16.3 |  |  |  |
| 56-65 | 16.9 | 18.4 |  |  |  |
| 66-84 | 23.5 | 30.2 |  |  |  |
| *University degree* |  |  | 83.33 |  | <0.001 |
| Yes | 36.0 | 40.8 |  |  |  |
| *Income* |  |  | 140.22 |  | <0.001 |
| Below poverty line^c^ | 20.7 | 25.9 |  |  |  |
| *Relationship status* |  |  | 19.04 |  | <0.001 |
| Living with partner | 63.5 | 65.7 |  |  |  |
| *Urbanicity* |  |  | 629.20 |  | <0.001 |
| Larger city | 30.8 | 43.5 |  |  |  |
| Smaller city or rural community | 69.2 | 56.5 |  |  |  |

^a^ Weighted percentages

^b^ Mean value for age in years only

^c^ Sixty percent of the national median equivalized disposable income

|  | Heterosexual born in Sweden | Heterosexual born outside of Europe | *X^2^* | F | Sig (p-value) |
| --- | --- | --- | --- | --- | --- |
| Total *n (%),* unweighted | 90 428 (86.4) | 4188 (4.0) |  |  |  |
|  | *%^a^ or M^b^* | *%^a^ or M^b^* |  |  |  |
| *Gender* |  |  | 20.18 |  | <0.001 |
| Male | 51.2 | 48.9 |  |  |  |
| Female | 48.8 | 51.1 |  |  |  |
| *Age, years (mean)* | 48.8 | 42.3 |  | 1184.30 | <0.001 |
| *Age, years* |  |  |  |  |  |
| 16-25 | 14.7 | 16.9 |  |  |  |
| 26-35 | 14.5 | 20.7 |  |  |  |
| 36-45 | 15.2 | 23.2 |  |  |  |
| 46-55 | 15.2 | 17.4 |  |  |  |
| 56-65 | 16.9 | 12.4 |  |  |  |
| 66-84 | 23.5 | 9.3 |  |  |  |
| *University degree* |  |  | 62.58 |  | <0.001 |
| Yes | 36.0 | 39.9 |  |  |  |
| *Income* |  |  | 2987.03 |  | 0.000 |
| Below poverty line^c^ | 20.7 | 44.3 |  |  |  |
| *Relationship status* |  |  | 267.97 |  | <0.001 |
| Living with partner | 63.5 | 55.4 |  |  |  |
| *Urbanicity* |  |  | 1479.00 |  | 0.000 |
| Larger city | 30.8 | 49.3 |  |  |  |
| Smaller city or rural community | 69.2 | 50.7 |  |  |  |

^a^ Weighted percentages

^b^ Mean value for age in years only

^c^ Sixty percent of the national median equivalized disposable income

|  | Heterosexual born in Sweden | | Sexual minority born in Sweden | | *X^2^* | | F | | Sig (p-value) | |
| --- | --- | --- | --- | --- | --- | --- | --- | --- | --- | --- |
| Total *n (%),* unweighted | 90 428 (86.4) | 2766 (2.6) | |  | |  | |  | |  |
|  | *%^a^ or M^b^* | *%^a^ or M^b^* | |  | |  | |  | |  |
| *Gender* |  |  | | 189.52 | |  | | <0.001 | |  |
| Male | 51.2 | 39.2 | |  | |  | |  | |  |
| Female | 48.8 | 60.8 | |  | |  | |  | |  |
| *Age, years (mean)* | 48.8 | 32.3 | |  | | 2547.17 | | 0.000 | |  |
| *Age, years* |  |  | |  | |  | |  | |  |
| 16-25 | 14.7 | 43.9 | |  | |  | |  | |  |
| 26-35 | 14.5 | 24.9 | |  | |  | |  | |  |
| 36-45 | 15.2 | 13.8 | |  | |  | |  | |  |
| 46-55 | 15.2 | 7.8 | |  | |  | |  | |  |
| 56-65 | 16.9 | 5.4 | |  | |  | |  | |  |
| 66-84 | 23.5 | 4.3 | |  | |  | |  | |  |
| *University degree* |  |  | | 11.99 | |  | | <0.001 | |  |
| Yes | 36.0 | 38.9 | |  | |  | |  | |  |
| *Income* |  |  | | 1545.15 | |  | | 0.000 | |  |
| Below poverty line^c^ | 20.7 | 48.9 | |  | |  | |  | |  |
| *Relationship status* |  |  | | 1083.62 | |  | | <0.001 | |  |
| Living with partner | 63.5 | 35.7 | |  | |  | |  | |  |
| *Urbanicity* |  |  | | 101.57 | |  | | <0.001 | |  |
| Larger city | 30.8 | 39.0 | |  | |  | |  | |  |
| Smaller city or rural community | 69.2 | 61.0 | |  | |  | |  | |  |

^a^ Weighted percentages

^b^ Mean value for age in years only

^c^ Sixty percent of the national median equivalized disposable income

|  | Heterosexual born in Sweden | Sexual minority born in Europe | *X^2^* | F | Sig (p-value) |
| --- | --- | --- | --- | --- | --- |
| Total *n (%),* unweighted | 90 428 (86.4) | 235 (0.2) |  |  |  |
|  | *%^a^ or M^b^* | *%^a^ or M^b^* |  |  |  |
| *Gender* |  |  | 1.99 |  | 0.158 |
| Male | 51.2 | 54.7 |  |  |  |
| Female | 48.8 | 45.3 |  |  |  |
| *Age, years (mean)* | 48.8 | 42.3 |  | 46.83 | <0.001 |
| *Age, years* |  |  |  |  |  |
| 16-25 | 14.7 | 15.0 |  |  |  |
| 26-35 | 14.5 | 32.7 |  |  |  |
| 36-45 | 15.2 | 15.5 |  |  |  |
| 46-55 | 15.2 | 11.5 |  |  |  |
| 56-65 | 16.9 | 10.6 |  |  |  |
| 66-84 | 23.5 | 14.7 |  |  |  |
| *University degree* |  |  | 32.32 |  | <0.001 |
| Yes | 36.0 | 49.6 |  |  |  |
| *Income* |  |  | 45.18 |  | <0.001 |
| Below poverty line^c^ | 20.7 | 34.2 |  |  |  |
| *Relationship status* |  |  | 17.77 |  | <0.001 |
| Living with partner | 63.5 | 53.3 |  |  |  |
| *Urbanicity* |  |  | 44.41 |  | <0.001 |
| Larger city | 30.8 | 46.2 |  |  |  |
| Smaller city or rural community | 69.2 | 53.8 |  |  |  |

^a^ Weighted percentages

^b^ Mean value for age in years only

^c^ Sixty percent of the national median equivalized disposable income

|  | Heterosexual born in Sweden | Sexual minority born outside of Europe | *X^2^* | F | Sig (p-value) |
| --- | --- | --- | --- | --- | --- |
| Total *n (%),* unweighted | 90 428 (86.4) | 233 (0.2) |  |  |  |
|  | *%^a^ or M^b^* | *%^a^ or M^b^* |  |  |  |
| *Gender* |  |  | 0.33 |  | 0.567 |
| Male | 51.2 | 50.0 |  |  |  |
| Female | 48.8 | 50.0 |  |  |  |
| *Age, years (mean)* | 48.8 | 34.4 |  | 347.54 | <0.001 |
| *Age, years* |  |  |  |  |  |
| 16-25 | 14.7 | 33.2 |  |  |  |
| 26-35 | 14.5 | 22.6 |  |  |  |
| 36-45 | 15.2 | 25.9 |  |  |  |
| 46-55 | 15.2 | 9.5 |  |  |  |
| 56-65 | 16.9 | 7.1 |  |  |  |
| 66-84 | 23.5 | 1.8 |  |  |  |
| *University degree* |  |  | 0.27 |  | 0.605 |
| Yes | 36.0 | 37.0 |  |  |  |
| *Income* |  |  | 441.44 |  | <0.001 |
| Below poverty line^c^ | 20.7 | 55.6 |  |  |  |
| *Relationship status* |  |  | 87.41 |  | <0.001 |
| Living with partner | 63.5 | 45.0 |  |  |  |
| *Urbanicity* |  |  | 213.28 |  | <0.001 |
| Larger city | 30.8 | 58.5 |  |  |  |
| Smaller city or rural community | 69.2 | 41.5 |  |  |  |

^a^ Weighted percentages

^b^ Mean value for age in years only

^c^ Sixty percent of the national median equivalized disposable income

|  | Heterosexual born in Europe | Heterosexual born outside of Europe | *X^2^* | F | Sig (p-value) |
| --- | --- | --- | --- | --- | --- |
| Total *n (%),* unweighted | 6802 (6.5) | 4188 (4.0) |  |  |  |
|  | *%^a^ or M^b^* | *%^a^ or M^b^* |  |  |  |
| *Gender* |  |  | 13.67 |  | <0.001 |
| Male | 46.3 | 48.9 |  |  |  |
| Female | 53.7 | 51.1 |  |  |  |
| *Age, years (mean)* | 53.2 | 42.3 |  | 2176.47 | 0.000 |
| *Age, years* |  |  |  |  |  |
| 16-25 | 6.5 | 16.9 |  |  |  |
| 26-35 | 12.9 | 20.7 |  |  |  |
| 36-45 | 15.7 | 23.2 |  |  |  |
| 46-55 | 16.3 | 17.4 |  |  |  |
| 56-65 | 18.4 | 12.4 |  |  |  |
| 66-84 | 30.2 | 9.3 |  |  |  |
| *University degree* |  |  | 1.61 |  | 0.205 |
| Yes | 40.8 | 39.9 |  |  |  |
| *Income* |  |  | 752.98 |  | <0.001 |
| Below poverty line^c^ | 25.9 | 44.3 |  |  |  |
| *Relationship status* |  |  | 228.34 |  | <0.001 |
| Living with partner | 65.7 | 55.4 |  |  |  |
| *Urbanicity* |  |  | 68.42 |  | <0.001 |
| Larger city | 43.5 | 49.3 |  |  |  |
| Smaller city or rural community | 56.5 | 50.7 |  |  |  |

^a^ Weighted percentages

^b^ Mean value for age in years only

^c^ Sixty percent of the national median equivalized disposable income

|  | Heterosexual born in Europe | Sexual minority born in Sweden | *X^2^* | F | Sig (p-value) |
| --- | --- | --- | --- | --- | --- |
| Total *n (%),* unweighted | 6802 (6.5) | 2766 (2.6) |  |  |  |
|  | *%^a^ or M^b^* | *%^a^ or M^b^* |  |  |  |
| *Gender* |  |  | 51.79 |  | <0.001 |
| Male | 46.3 | 39.2 |  |  |  |
| Female | 53.7 | 60.8 |  |  |  |
| *Age, years (mean)* | 53.2 | 32.3 |  | 3855.87 | 0.000 |
| *Age, years* |  |  |  |  |  |
| 16-25 | 6.5 | 43.9 |  |  |  |
| 26-35 | 12.9 | 24.9 |  |  |  |
| 36-45 | 15.7 | 13.8 |  |  |  |
| 46-55 | 16.3 | 7.8 |  |  |  |
| 56-65 | 18.4 | 5.4 |  |  |  |
| 66-84 | 30.2 | 4.3 |  |  |  |
| *University degree* |  |  | 3.63 |  | 0.057 |
| Yes | 40.8 | 38.9 |  |  |  |
| *Income* |  |  | 617.53 |  | <0.001 |
| Below poverty line^c^ | 25.9 | 48.9 |  |  |  |
| *Relationship status* |  |  | 936.70 |  | <0.001 |
| Living with partner | 65.7 | 35.7 |  |  |  |
| *Urbanicity* |  |  | 21.46 |  | <0.001 |
| Larger city | 43.5 | 39.0 |  |  |  |
| Smaller city or rural community | 56.5 | 61.0 |  |  |  |

^a^ Weighted percentages

^b^ Mean value for age in years only

^c^ Sixty percent of the national median equivalized disposable income

|  | Heterosexual born in Europe | Sexual minority born in Europe | *X^2^* | F | Sig (p-value) |
| --- | --- | --- | --- | --- | --- |
| Total *n (%),* unweighted | 6802 (6.5) | 235 (0.2) |  |  |  |
|  | *%^a^ or M^b^* | *%^a^ or M^b^* |  |  |  |
| *Gender* |  |  | 10.99 |  | <0.001 |
| Male | 46.3 | 54.7 |  |  |  |
| Female | 53.7 | 45.3 |  |  |  |
| *Age, years (mean)* | 53.2 | 42.3 |  | 148.47 | <0.001 |
| *Age, years* |  |  |  |  |  |
| 16-25 | 6.5 | 15.0 |  |  |  |
| 26-35 | 12.9 | 32.7 |  |  |  |
| 36-45 | 15.7 | 15.5 |  |  |  |
| 46-55 | 16.3 | 11.5 |  |  |  |
| 56-65 | 18.4 | 10.6 |  |  |  |
| 66-84 | 30.2 | 14.7 |  |  |  |
| *University degree* |  |  | 12.57 |  | <0.001 |
| Yes | 40.8 | 49.6 |  |  |  |
| *Income* |  |  | 13.95 |  | <0.001 |
| Below poverty line^c^ | 25.9 | 34.2 |  |  |  |
| *Relationship status* |  |  | 26.27 |  | <0.001 |
| Living with partner | 65.7 | 53.3 |  |  |  |
| *Urbanicity* |  |  | 1.13 |  | 0.287 |
| Larger city | 43.5 | 46.2 |  |  |  |
| Smaller city or rural community | 56.5 | 53.8 |  |  |  |

^a^ Weighted percentages

^b^ Mean value for age in years only

^c^ Sixty percent of the national median equivalized disposable income

|  | Heterosexual born in Europe | Sexual minority born outside of Europe | *X^2^* | F | Sig (p-value) |
| --- | --- | --- | --- | --- | --- |
| Total *n (%),* unweighted | 6802 (6.5) | 233 (0.2) |  |  |  |
|  | *%^a^ or M^b^* | *%^a^ or M^b^* |  |  |  |
| *Gender* |  |  | 3.12 |  | 0.077 |
| Male | 46.3 | 50.0 |  |  |  |
| Female | 53.7 | 50.0 |  |  |  |
| *Age, years (mean)* | 53.2 | 34.4 |  | 662.78 | <0.001 |
| *Age, years* |  |  |  |  |  |
| 16-25 | 6.5 | 33.2 |  |  |  |
| 26-35 | 12.9 | 22.6 |  |  |  |
| 36-45 | 15.7 | 25.9 |  |  |  |
| 46-55 | 16.3 | 9.5 |  |  |  |
| 56-65 | 18.4 | 7.1 |  |  |  |
| 66-84 | 30.2 | 1.8 |  |  |  |
| *University degree* |  |  | 3.28 |  | 0.070 |
| Yes | 40.8 | 37.0 |  |  |  |
| *Income* |  |  | 250.32 |  | <0.001 |
| Below poverty line^c^ | 25.9 | 55.6 |  |  |  |
| *Relationship status* |  |  | 106.01 |  |  |
| Living with partner | 65.7 | 45.0 |  |  |  |
| *Urbanicity* |  |  | 51.48 |  | <0.001 |
| Larger city | 43.5 | 58.5 |  |  |  |
| Smaller city or rural community | 56.5 | 41.5 |  |  |  |

^a^ Weighted percentages

^b^ Mean value for age in years only

^c^ Sixty percent of the national median equivalized disposable income

|  | Heterosexual born outside of Europe | Sexual minority born in Sweden | *X^2^* | F | Sig (p-value) |
| --- | --- | --- | --- | --- | --- |
| Total *n (%),* unweighted | 4188 (4.0) | 2766 (2.6) |  |  |  |
|  | *%^a^ or M^b^* | *%^a^ or M^b^* |  |  |  |
| *Gender* |  |  | 98.81 |  | <0.001 |
| Male | 48.9 | 39.2 |  |  |  |
| Female | 51.1 | 60.8 |  |  |  |
| *Age, years (mean)* | 42.3 | 32.3 |  | 1040.94 | <0.001 |
| *Age, years* |  |  |  |  |  |
| 16-25 | 16.9 | 43.9 |  |  |  |
| 26-35 | 20.7 | 24.9 |  |  |  |
| 36-45 | 23.2 | 13.8 |  |  |  |
| 46-55 | 17.4 | 7.8 |  |  |  |
| 56-65 | 12.4 | 5.4 |  |  |  |
| 66-84 | 9.3 | 4.3 |  |  |  |
| *University degree* |  |  | 1.07 |  | 0.301 |
| Yes | 39.9 | 38.9 |  |  |  |
| *Income* |  |  | 22.67 |  | <0.001 |
| Below poverty line^c^ | 44.3 | 48.9 |  |  |  |
| *Relationship status* |  |  | 406.00 |  | <0.001 |
| Living with partner | 55.4 | 35.7 |  |  |  |
| *Urbanicity* |  |  | 112.06 |  | <0.001 |
| Larger city | 49.3 | 39.0 |  |  |  |
| Smaller city or rural community | 50.7 | 61.0 |  |  |  |

^a^ Weighted percentages

^b^ Mean value for age in years only

^c^ Sixty percent of the national median equivalized disposable income

|  | Heterosexual born outside of Europe | Sexual minority born in Europe | *X^2^* | F | Sig (p-value) |
| --- | --- | --- | --- | --- | --- |
| Total *n (%),* unweighted | 4188 (4.0) | 235 (0.2) |  |  |  |
|  | *%^a^ or M^b^* | *%^a^ or M^b^* |  |  |  |
| *Gender* |  |  | 5.27 |  | 0.022 |
| Male | 48.9 | 54.7 |  |  |  |
| Female | 51.1 | 45.3 |  |  |  |
| *Age, years (mean)* | 42.3 | 42.3 |  | 0.009 | 0.924 |
| *Age, years* |  |  |  |  |  |
| 16-25 | 16.9 | 15.0 |  |  |  |
| 26-35 | 20.7 | 32.7 |  |  |  |
| 36-45 | 23.2 | 15.5 |  |  |  |
| 46-55 | 17.4 | 11.5 |  |  |  |
| 56-65 | 12.4 | 10.6 |  |  |  |
| 66-84 | 9.3 | 14.7 |  |  |  |
| *University degree* |  |  | 15.35 |  | <0.001 |
| Yes | 39.9 | 49.6 |  |  |  |
| *Income* |  |  | 16.09 |  | <0001 |
| Below poverty line^c^ | 44.3 | 34.2 |  |  |  |
| *Relationship status* |  |  | 0.66 |  | 0.417 |
| Living with partner | 55.4 | 53.3 |  |  |  |
| *Urbanicity* |  |  | 1.49 |  | 0.222 |
| Larger city | 49.3 | 46.2 |  |  |  |
| Smaller city or rural community | 50.7 | 53.8 |  |  |  |

^a^ Weighted percentages

^b^ Mean value for age in years only

^c^ Sixty percent of the national median equivalized disposable income

|  | Heterosexual born outside of Europe | Sexual minority born outside of Europe | *X^2^* | F | Sig (p-value) |
| --- | --- | --- | --- | --- | --- |
| Total *n (%),* unweighted | 4188 (4.0) | 233 (0.2) |  |  |  |
|  | *%^a^ or M^b^* | *%^a^ or M^b^* |  |  |  |
| *Gender* |  |  | 0.29 |  | 0.593 |
| Male | 48.9 | 50.0 |  |  |  |
| Female | 51.1 | 50.0 |  |  |  |
| *Age, years (mean)* | 42.3 | 34.4 |  | 139.54 | <0.001 |
| *Age, years* |  |  |  |  |  |
| 16-25 | 16.9 | 33.2 |  |  |  |
| 26-35 | 20.7 | 22.6 |  |  |  |
| 36-45 | 23.2 | 25.9 |  |  |  |
| 46-55 | 17.4 | 9.5 |  |  |  |
| 56-65 | 12.4 | 7.1 |  |  |  |
| 66-84 | 9.3 | 1.8 |  |  |  |
| *University degree* |  |  | 1.96 |  | 0.162 |
| Yes | 39.9 | 37.0 |  |  |  |
| *Income* |  |  | 29.72 |  | <0.001 |
| Below poverty line^c^ | 44.3 | 55.6 |  |  |  |
| *Relationship status* |  |  | 24.72 |  | <0.001 |
| Living with partner | 55.4 | 45.0 |  |  |  |
| *Urbanicity* |  |  | 19.35 |  | <0.001 |
| Larger city | 49.3 | 58.5 |  |  |  |
| Smaller city or rural community | 50.7 | 41.5 |  |  |  |

^a^ Weighted percentages

^b^ Mean value for age in years only

^c^ Sixty percent of the national median equivalized disposable income

|  | Sexual minority born in Sweden | Sexual minority born in Europe | *X^2^* | F | Sig (p-value) |
| --- | --- | --- | --- | --- | --- |
| Total *n (%),* unweighted | 2766 (2.6) | 235 (0.2) |  |  |  |
|  | *%^a^ or M^b^* | *%^a^ or M^b^* |  |  |  |
| *Gender* |  |  | 36.03 |  | <0.001 |
| Male | 39.2 | 54.7 |  |  |  |
| Female | 60.8 | 45.3 |  |  |  |
| *Age, years (mean)* | 32.3 | 42.3 |  | 158.84 | <0.001 |
| *Age, years* |  |  |  |  |  |
| 16-25 | 43.9 | 15.0 |  |  |  |
| 26-35 | 24.9 | 32.7 |  |  |  |
| 36-45 | 13.8 | 15.5 |  |  |  |
| 46-55 | 7.8 | 11.5 |  |  |  |
| 56-65 | 5.4 | 10.6 |  |  |  |
| 66-84 | 4.3 | 14.7 |  |  |  |
| *University degree* |  |  | 17.31 |  | <0.001 |
| Yes | 38.9 | 49.6 |  |  |  |
| *Income* |  |  | 31.44 |  | <0.001 |
| Below poverty line^c^ | 48.9 | 34.2 |  |  |  |
| *Relationship status* |  |  | 48.04 |  | <0.001 |
| Living with partner | 35.7 | 53.3 |  |  |  |
| *Urbanicity* |  |  | 7.90 |  | 0.005 |
| Larger city | 39.0 | 46.2 |  |  |  |
| Smaller city or rural community | 61.0 | 53.8 |  |  |  |

^a^ Weighted percentages

^b^ Mean value for age in years only

^c^ Sixty percent of the national median equivalized disposable income

|  | Sexual minority born in Sweden | Sexual minority born outside of Europe | *X^2^* | F | Sig (p-value) |
| --- | --- | --- | --- | --- | --- |
| Total *n (%),* unweighted | 2766 (2.6) | 233 (0.2) |  |  |  |
|  | *%^a^ or M^b^* | *%^a^ or M^b^* |  |  |  |
| *Gender* |  |  | 24.75 |  | <0.001 |
| Male | 39.2 | 50.0 |  |  |  |
| Female | 60.8 | 50.0 |  |  |  |
| *Age, years (mean)* | 32.3 | 34.4 |  | 10.24 | 0.001 |
| *Age, years* |  |  |  |  |  |
| 16-25 | 43.9 | 33.2 |  |  |  |
| 26-35 | 24.9 | 22.6 |  |  |  |
| 36-45 | 13.8 | 25.9 |  |  |  |
| 46-55 | 7.8 | 9.5 |  |  |  |
| 56-65 | 5.4 | 7.1 |  |  |  |
| 66-84 | 4.3 | 1.8 |  |  |  |
| *University degree* |  |  | 0.76 |  | 0.383 |
| Yes | 38.9 | 37.0 |  |  |  |
| *Income* |  |  | 9.26 |  | 0.002 |
| Below poverty line^c^ | 48.9 | 55.6 |  |  |  |
| *Relationship status* |  |  | 19.06 |  | <0.001 |
| Living with partner | 35.7 | 45.0 |  |  |  |
| *Urbanicity* |  |  | 80.26 |  | <0.001 |
| Larger city | 39.0 | 58.5 |  |  |  |
| Smaller city or rural community | 61.0 | 41.5 |  |  |  |

^a^ Weighted percentages

^b^ Mean value for age in years only

^c^ Sixty percent of the national median equivalized disposable income

|  | Sexual minority born in Europe | Sexual minority born outside of Europe | *X^2^* | F | Sig (p-value) |
| --- | --- | --- | --- | --- | --- |
| Total *n (%),* unweighted | 235 (0.2) | 233 (0.2) |  |  |  |
|  | *%^a^ or M^b^* | *%^a^ or M^b^* |  |  |  |
| *Gender* |  |  | 2.13 |  | 0.145 |
| Male | 54.7 | 50.0 |  |  |  |
| Female | 45.3 | 50.0 |  |  |  |
| *Age, years (mean)* | 42.3 | 34.4 |  | 68.83 | <0.001 |
| *Age, years* |  |  |  |  |  |
| 16-25 | 15.0 | 33.2 |  |  |  |
| 26-35 | 32.7 | 22.6 |  |  |  |
| 36-45 | 15.5 | 25.9 |  |  |  |
| 46-55 | 11.5 | 9.5 |  |  |  |
| 56-65 | 10.6 | 7.1 |  |  |  |
| 66-84 | 14.7 | 1.8 |  |  |  |
| *University degree* |  |  | 15.73 |  | <0.001 |
| Yes | 49.6 | 37.0 |  |  |  |
| *Income* |  |  | 44.62 |  | <0.001 |
| Below poverty line^c^ | 34.2 | 55.6 |  |  |  |
| *Relationship status* |  |  | 6.71 |  | 0.010 |
| Living with partner | 53.3 | 45.0 |  |  |  |
| *Urbanicity* |  |  | 14.72 |  | <0.001 |
| Larger city | 46.2 | 58.5 |  |  |  |
| Smaller city or rural community | 53.8 | 41.5 |  |  |  |

^a^ Weighted percentages

^b^ Mean value for age in years only

^c^ Sixty percent of the national median equivalized disposable income
