# Supplementary material for: Differences in mental health symptoms and treatment by sexual orientation and migration background in a population-based sample
Source: Soc Psychiatry Psychiatr Epidemiol. 2025 Feb 17;60(5):1197–209. doi: 10.1007/s00127-025-02848-w (PMC12119674; doi:10.1007/s00127-025-02848-w)
Supplement: Supplementary file 2 — Supplementary file2 (DOCX 24 kb) [file 127_2025_2848_MOESM2_ESM.docx]

**Online Resource 2**

**Title** Differences in mental health symptoms and treatment by sexual orientation and migration background in a population-based sample

**Journal** Social Psychiatry and Psychiatric Epidemiology

**Authors** Andreas Malm, Petter Tinghög, Richard Bränström

**Corresponding author** Andreas Malm ([andreas.malm@ki.se](mailto:andreas.malm@ki.se)), Department of Clinical Neuroscience, Karolinska Institutet, Nobels väg 9, 171 77 Stockholm, Sweden

| **Online Resource 2** Interaction of sexual orientation and interpersonal and social stress on mental health symptoms and treatment for common mental disorders, and interaction of migration status and interpersonal and social stress on mental health symptoms and treatment for common mental disorders | | | | | |
| --- | --- | --- | --- | --- | --- |
|  | Mental health symptoms  Adjusted ß | |  | Treatment for common mental disorders  Adjusted ß | |
| *Main effects^a^* |  |  |  |  |  |
| Discrimination | .873* |  |  | .527* |  |
| Victimization | .371* |  |  | .343* |  |
| Social support | .754* |  |  | .188* |  |
| Social trust | .498* |  |  | .330* |  |
|  |  |  |  |  |  |
| *Sexual orientation as effect modifier^b^* |  |  |  |  |  |
| Discrimination x sexual orientation | -.194 |  |  | -.040 |  |
| Victimization x sexual orientation | .016 |  |  | .139 |  |
| Social support x sexual orientation | -.403* |  |  | -.133 |  |
| Social trust x sexual orientation | .105 |  |  | .333* |  |
|  |  |  |  |  |  |
| *Migration status as effect modifier^c^* |  |  |  |  |  |
| Discrimination x European born | .000 |  |  | -.198 |  |
| Discrimination x non-European born | -.192 |  |  | .064 |  |
| Victimization x European born | .279 |  |  | .064 |  |
| Victimization x non-European born | .103 |  |  | .299 |  |
| Social support x European born | .054 |  |  | -.170 |  |
| Social support x non-European born | -.189 |  |  | .022 |  |
| Social trust x European born | .036 |  |  | -.042 |  |
| Social trust x non-European born | -.056 |  |  | -.383* |  |
|  |  |  |  |  |  |
|  | | | | | |

* Significant at *p* < 0.05
^a^ Models adjusted for gender, age, level of education, income, marital status, urbanicity, migration status, and sexual orientation
^b^ Models adjusted for gender, age, level of education, income, marital status, urbanicity, and migration status
^c^ Models adjusted for gender, age, level of education, income, marital status, urbanicity, and sexual orientation
